# Genetically Modified and Gene-Edited Organisms—Objectives, Public Perception and Applications

**DOI:** 10.3390/biology15171563

**Published:** 2026-09-07

**Authors:** László Solti, Hedvig Fébel

**Affiliations:** Department of Obstetrics and Food Animal Medicine Clinic, University of Veterinary Medicine, 1078 Budapest, Hungary; febel.hedvig@univet.hu

**Keywords:** genetic modification, genome editing, transgenic, human medicine, practical use, public perception, new EU legislation, acceptance, rejection

## Abstract

Genetic modification and genome editing have become important tools in agriculture, animal production, biotechnology, and human medicine, but their safety and societal acceptance remain subjects of debate. This review examines genetically modified (GM) and gene-edited organisms, distinguishing transgenesis from precision genome editing technologies, including CRISPR/Cas9, base editing, and prime editing. Representative applications in crops, livestock, pharmaceutical production, and xenotransplantation are discussed, together with their regulatory framework and public perception. Current scientific assessments indicate that approved GM foods are not inherently more hazardous to human health than their conventional counterparts when evaluated case by case. Potential benefits include improved nutritional quality, biofortification, disease resistance, increased agricultural efficiency, production of therapeutic proteins, and applications in animal health and medicine. Possible concerns include allergenicity, toxicity, unintended genetic or phenotypic effects, altered nutritional composition, environmental consequences, animal welfare issues, and uncertainties associated with long-term or large-scale deployment. Public acceptance varies substantially according to geographical region, application, cultural and ethical considerations, regulatory environment, scientific literacy, and institutional trust. Overall, GM and gene-edited organisms should not be considered a homogeneous category. Their benefits, risks, and societal acceptability depend on the specific organism, genetic modification, intended trait, and context of use, supporting a balanced, evidence-based, and case-specific approach.

## Simple Summary

The debate about the acceptance or rejection of genetically modified organisms (GMOs) has lasted for many decades. At the heart of this debate is the balance between the potential benefits of GMOs and concerns about their possible effects on human health, the environment, animal welfare and society. Over the years, several plants, animals and raw materials have been produced by GMOs for pharmaceutical, medical, industrial and agricultural purposes. Some of them became accepted and introduced into daily practice, while others were refused, especially those for human alimentation. Recently, however, since the invention of new-generation gene editing methods, there is a need to reconsider and redefine the term GMO as well as the acceptance of products created in this way.

## 1. Introduction

Genetic modification of living organisms is not a recent invention. Humans have selected spontaneously occurring mutants of plants and animals with advantageous properties for thousands of years to improve crops, livestock, and food; artificial selection of wolves began roughly 30,000 years ago, and the oldest evidence of crop selection dates back to 7800 BC. Mutation has also long been induced deliberately using strong chemicals or irradiation. In today’s usage, the term “genetically modified organism” (GMO) refers more narrowly to organisms whose genetic material has been altered by genetic engineering—a decades-old approach that is increasingly complemented and, in some applications, replaced by targeted genome editing. The definition of what counts as a GMO is not merely semantic: whether organisms produced by genome editing, a technique introduced only about a decade ago, are legally treated as GMOs determines whether existing regulatory restrictions apply to them. According to a 2018 ruling of the Court of Justice of the European Union, organisms obtained through gene editing technology are legally regarded as genetically modified, which has, until recently, placed considerable restrictions on their use within the EU.

Since the first genetically modified bacterium was produced in 1973, genetic modification has moved from basic molecular biology into medicine, agriculture, and industry, generating products ranging from recombinant human insulin to disease-resistant crops and livestock, biopharmaceuticals produced in the milk or eggs of transgenic animals, and genetically modified pigs developed as organ donors for xenotransplantation. At the same time, few areas of modern biotechnology have generated as much sustained public debate as GMOs. While scientific assessments generally indicate that approved GM products are as safe as their conventional counterparts, societal acceptance of GMOs varies considerably across continents, countries, product types, and demographic groups and is shaped by ethical, cultural, regulatory, and communication-related factors that are largely independent of the underlying scientific evidence. Regulatory frameworks reflect this divide: some jurisdictions permit genetically modified products with minimal restriction, others prohibit their cultivation while allowing import, and a small number ban them altogether. The European Union’s recent adoption of a risk-tiered regulatory framework for new genomic techniques (NGT-1 and NGT-2 categories) illustrates how this regulatory landscape continues to evolve alongside the technology itself.

Given the breadth and depth of the literature on genetic modification, an exhaustive review of this field is beyond the scope of a single article. Rather than attempting complete coverage, this review aims to provide a structured overview of the field by (i) clarifying the terminology and methods used to distinguish conventional breeding, transgenesis, and genome, base, and prime editing; (ii) presenting selected, representative examples of genetically modified organisms across several major areas of application, including crops, disease-resistant livestock, food-producing animals, pharmaceutical production (“gene pharming”), and organ donor pigs for xenotransplantation; (iii) summarizing the global regulatory landscape governing genetically modified and gene-edited organisms; and (iv) describing the current state of public perception and the ongoing debate surrounding the acceptance and rejection of GMOs. In doing so, this review seeks to bring together the scientific, regulatory, and societal dimensions of genetic modification within a single, coherent framework.

The relevant scientific literature was identified through systematic searches of PubMed/MEDLINE, Web of Science, Scopus and Google Scholar. Additional relevant publications were identified by screening the reference lists of selected articles and by using citation-tracking tools to identify papers that cited key publications. The literature search focused on studies addressing GMOs, their applications, potential environmental and health impacts, and related regulatory and societal aspects.

## 2. Methods of Genetic Modification

### 2.1. Conventional Breeding

This method relies on crossing parents. Farmers have been altering plants’ genetics for thousands of years using conventional techniques, such as selection and crossbreeding. This process has gradually transformed wild species into the crops we grow today. Spontaneous mutants with desirable traits were selected and used for crossbreeding which combines traits from two different parents of the same (or closely related) species through sexual hybridization. Selection and backcrossing: Progeny are repeatedly crossed back to an elite parent line over multiple generations to isolate the target trait while removing unwanted characteristics. Later, random DNA breaks and changes (mutations) were provoked by physical and chemical treatments or irradiation. Conventional breeding and mutagenesis are foundational improvement strategies used to accelerate genetic variation and enhance traits. These methods, however, never introduce foreign DNA, distinguishing them from modern transgenic GMO methods.

### 2.2. Genetically Modified Organisms (GMOs)

Genetically modified organisms (GMOs) have their own genome altered (by adding, removing, or changing specific genes) by genetic engineering. In this process, no foreign DNA is introduced into the organism, creating desired traits like pest resistance in crops or medicine production in bacteria. Examples: Flavr Savr tomatoes, Non-Browning Apples (Arctic Apples).

### 2.3. Transgenic Organisms

Transgenic organisms are GMOs that have been genetically modified to contain DNA from different species, resulting in the expression of traits not naturally found in those organisms. Scientists transfer a whole gene from one species into an entirely different species (e.g., placing soil bacteria genes into corn). The gene is inserted randomly into the host genome, which requires years of screening to ensure it works correctly without disrupting other traits. Indeed, many GMOs contain genes from unrelated species, which allows the GMOs to have traits that would be difficult or impossible to develop through traditional selective breeding. This foreign DNA is introduced using techniques such as gene cloning, recombinant DNA technology, or genetic engineering. Examples: golden rice, Bt cotton [1].

### 2.4. Genome Editing

Genome editing is defined as a technique used to change the structure of a gene or genome through engineered nucleases, allowing for the insertion, replacement, or removal of genes. This process involves creating double-strand breaks at specific locations in the genome, which are then repaired by the cell’s endogenous mechanisms. Key methods include CRISPR-Cas9, TALENs, and Zinc-finger nucleases. These tools act like molecular scissors to cut DNA at exact spots so pieces can be added, removed, or changed without adding foreign DNA. Instead, they act like a genetic “find-and-replace” tool to tweak, delete, or turn off genes that already exist inside the organism’s own DNA. This process is highly precise, much faster, and mimics mutations that could happen naturally over time.

CRISPR/Cas9 is used for programmed genome editing, as the system allows more precise modification of genes than ever before—therefore it is called targeted mutagenesis or precision breeding. The method was copied from the defense system of bacteria, for which its inventors (Jennifer A. Doudna and Emmanuelle Charpentier) were awarded the Nobel Prize in 2020. Bacteria protect themselves against viral infections with the help of short, repetitive DNA segments in their genomes (Clustered Regularly Interspaced Short Palindromic Repeats, CRISPR). Each repeat segment is followed by a short “spacer” DNA sequence that corresponds to a segment of a virus or plasmid that the bacterium has previously encountered. Cas9, on the other hand, is a DNA-cutting enzyme that recognizes foreign nucleic acids that enter the bacterial cell based on their spacers and cuts them into pieces. Gene editing is used as a biological version of the “find and replace” function of text editors: if a “guide RNA” and the genetic scissors (Cas9 enzyme) are introduced into the cell, the enzyme cleaves its DNA at a specific location. Using a synthetic guide RNA molecule, Cas9 will cut both strands of the cell’s DNA at the desired point. The DNA break is patched up by the cell’s self-repair mechanisms, but spontaneous repair usually leads to disruption of the gene in question (this is a gene knockout). However, if a specially designed template (“repair sequence”) is introduced into the system, it will replace the cut DNA section [2]. By this procedure, a lot of gene-edited plants and animals have been produced in the last few years. Despite the severe ethical and biological concerns about the use of this method on human eggs, genetically modified children have already been born in China. A scientist Jiankui He, claimed to have “created” the first gene-edited babies, designed to be naturally immune to the human immunodeficiency virus (HIV). The news immediately triggered widespread criticism, denouncement, and debate over the scientific and ethical legitimacy of his genetic experiments. The first genetically gene-edited babies were condemned saying: It’s “irresponsible and too early” [3]. He and two collaborators have been found guilty of conducting "illegal medical practices" and sentenced to 3 years in prison [4]. China’s guidelines and regulations have banned germline genome editing on human embryos for clinical use because of scientific and ethical concerns, in accordance with the international consensus [5].

CRISPR indeed revolutionized gene editing by enabling a precision level previously unattainable. Yet, technology is not without limitations: aneuploidy, which is defined as an abnormal number of chromosomes, was an unintended side effect of editing embryos with CRISPR. Even though CRISPR improved upon older genome editing technologies, it is not perfect. For example, sometimes genome editing tools cut in the wrong spot. Scientists are not yet sure how these errors might affect patients.

### 2.5. Base Editing

Base editing is an advanced genetic tool that changes single DNA letters without breaking both strands of DNA. The two main types are cytosine base editors and adenine base editors, which allow for the fixing of specific point mutations tied to inherited diseases. Unlike standard CRISPR, it avoids cutting the main DNA backbone, lowering the risk of random errors or large insertions and deletions [6].

It is an advanced and highly precise tool that allows for targeted genome modifications without cutting the DNA’s double helix, thus avoiding the toxic byproducts and unintended mutations often seen with CRISPR-Cas9 [7]. Base editing swaps individual DNA letters (A, T, C, G) with precise point mutations, directly addressing genetic diseases. Unlike traditional CRISPR-Cas9, base editing uses an enzyme to change a single target letter without breaking the DNA backbone, making it much safer. So far, two classes of DNA base editors have been described, cytosine base editors and adenine base editors. Base editing allows for the direct conversion of one single DNA base (or “letter”) into another without creating double-strand breaks in the genome. It fuses a modified Cas9 protein with an enzyme like a deaminase. This enzyme changes the chemical structure of the target base, converting it into a different base. This is ideal for correcting single nucleotide polymorphisms responsible for various genetic diseases.

Related to food products from gene-edited plants and animals, the European Food Safety Authority (EFSA) panel conducted a meta-analysis and reported to the European Commission that the new genomic techniques, including base editing and prime editing, did not reveal new hazards or risks not previously considered in EFSA scientific opinions. Off-target mutations from genome editing are similar in nature to those from conventional breeding and do not pose novel hazards. Consequently, based on the currently available data, no new potential hazards, and thus no new risks to humans, animals, or the environment, have been identified [8,9].

Recently, Columbia University released a preprint demonstrating the use of base editing that did not cause aneuploidy in treated human embryos [10]. However, it still had significant off-target effects, e.g., editing at sites other than the gene of interest, and cases of mosaic embryos, in which not all cells in the embryo carry the gene edits. For these reasons, more work and ethics discussions are necessary before using base-edited embryos for clinical IVF. There is a consensus that the genomes of germline cells must not be edited at this time because edits to a germline cell would be passed down through generations; many countries and organizations have strict regulations to prevent germline editing for this reason, and the NIH, for example, does not fund research to edit human embryos.

### 2.6. Prime Editing

Prime editing is a “search-and-replace” genome editing technology that directly writes new genetic information into a targeted DNA site. It uses a Cas9 nickase fused to a reverse transcriptase and a specialized prime editing guide RNA (pegRNA). It makes precise insertions, deletions, and base swaps without double-strand breaks. By avoiding the cutting of both DNA strands, it reduces toxic cell stress and major chromosomal errors and does not require extra external DNA donor templates to paste new segments [11].

## 3. Historical Milestones

The development of modern genetic modification can be traced through a series of foundational discoveries (Table 1). Following the 1953 description of the DNA double helix by Watson and Crick, Cohen and Boyer created the first recombinant DNA organism in 1973 by inserting a kanamycin-resistance gene into a bacterial plasmid and transferring it into another bacterium [12]. This breakthrough soon enabled the development of a modified *E. coli* strain producing human insulin, commercialized as Humulin in 1982 by Eli Lilly following research by Genentech—the first consumer GMO product approved by the U.S. FDA [13].

The first genetically modified animal was produced in 1974 when all tissues of a GM mouse were shown to contain an introduced foreign gene. The first genetically modified plant, an antibiotic-resistant tobacco, followed in 1983, and in 1994 the FDA approved the Flavr Savr tomato, the first commercially grown, genetically modified whole food. Insect-resistant Bt corn and herbicide-tolerant Roundup Ready soybeans reached commercial planting in 1995/1996, and in 2000 the first nutritionally enhanced GM food plant, golden rice, was produced. The 2010s ushered in the gene editing era: Doudna, Charpentier and colleagues published the CRISPR-Cas9 system in 2012, work recognized with the Nobel Prize in 2020; base editing was introduced in 2016 and prime editing in 2019, both of which avoid double-strand DNA breaks associated with standard CRISPR-Cas9. In 2015, AquAdvantage salmon became the first genetically engineered animal approved by the FDA for human consumption, and in 2020 the FDA approved GalSafe pigs, engineered to lack the alpha-gal sugar responsible for a meat allergy and for hyperacute transplant rejection.

## 4. Applications of Genetic Modification

The purposes of genetic modification are diverse. Many fall within the scope of human medicine, such as the production of pharmaceutical raw materials, the acquisition of organs and tissues for xenotransplantation, and the creation of animal models for studying human diseases. In crop and livestock breeding, the objective is to improve production or quality traits, to increase resistance to diseases or pests, and, in industry, to produce new consumer or industrial products. As the literature on this topic is vast, this review does not aim for exhaustive coverage but instead presents selected examples from several major areas of application. Some of these examples have been successfully introduced into everyday practice, while others—for biological, commercial, or regulatory reasons, and in some cases because of societal rejection—have not yet achieved their intended outcomes (Table 2).

### 4.1. Genetically Modified Microorganisms

Because of their simple structure, bacteria were the first in which the genetic code was modified: in 1973, Boyer and Cohen invented recombinant DNA technology by inserting kanamycin-resistance gene from one bacterium into a bacterial DNA ring called plasmid, and then this gene construct was transformed to another bacterium [12].

The practical breakthrough in genetic engineering soon allowed for the development of modified *E. coli* strain containing the human insulin gene. GM bacteria make human insulin by taking the human insulin gene, putting it into a plasmid, and growing the modified bacteria in large tanks to mass-produce the identical human hormone. These bacteria produce human insulin for diabetic patients, which was commercialized in 1982 by Eli Lilly following research breakthroughs by Genentech. Humulin became the first consumer GMO product approved by the U.S. Food and Drug Administration (FDA) [13].

Earlier, insulin was extracted from the pancreas of slaughterhouse pigs—200 g of purified insulin required nearly 2 tons of pancreas, and each batch was slightly different from the others. And in some cases, even though pig insulin differs from human insulin in only one amino acid, the drug caused an immune reaction in treated patients as a foreign substance. Humulin, produced by GM bacteria—cheaper than before—was the first registered insulin preparation that had no side effects. Humulin, produced by GM bacteria—cheaper than before—was the first registered insulin preparation that had no side effects [41].

Blood coagulation factors, interferon, erythropoietin, tissue plasminogen activator and growth hormone are also produced by GM bacteria. Extracting and purifying medicinal proteins produced in bacteria is not easy; however, they are significantly safer than any previous preparations obtained from cadavers, which inevitably involved the risk of infection with AIDS, hepatitis C and the Creutzfeldt–Jakob syndrome. Various GM microorganisms are also used to produce enzymes used in food processing, such as alpha amylase to break down starch into sugars, chymosin used in cheese production, and pectinesterase to improve the purity of fruit juices.

Recently, enhanced Genetically Modified Microorganisms can synthesize complex alternative proteins, animal-free dairy ingredients, and specialized functional enzymes for precision fermentation utilizing engineered yeast and fungal strains [42].

### 4.2. Genetically Modified Crops

**Figure 1:**
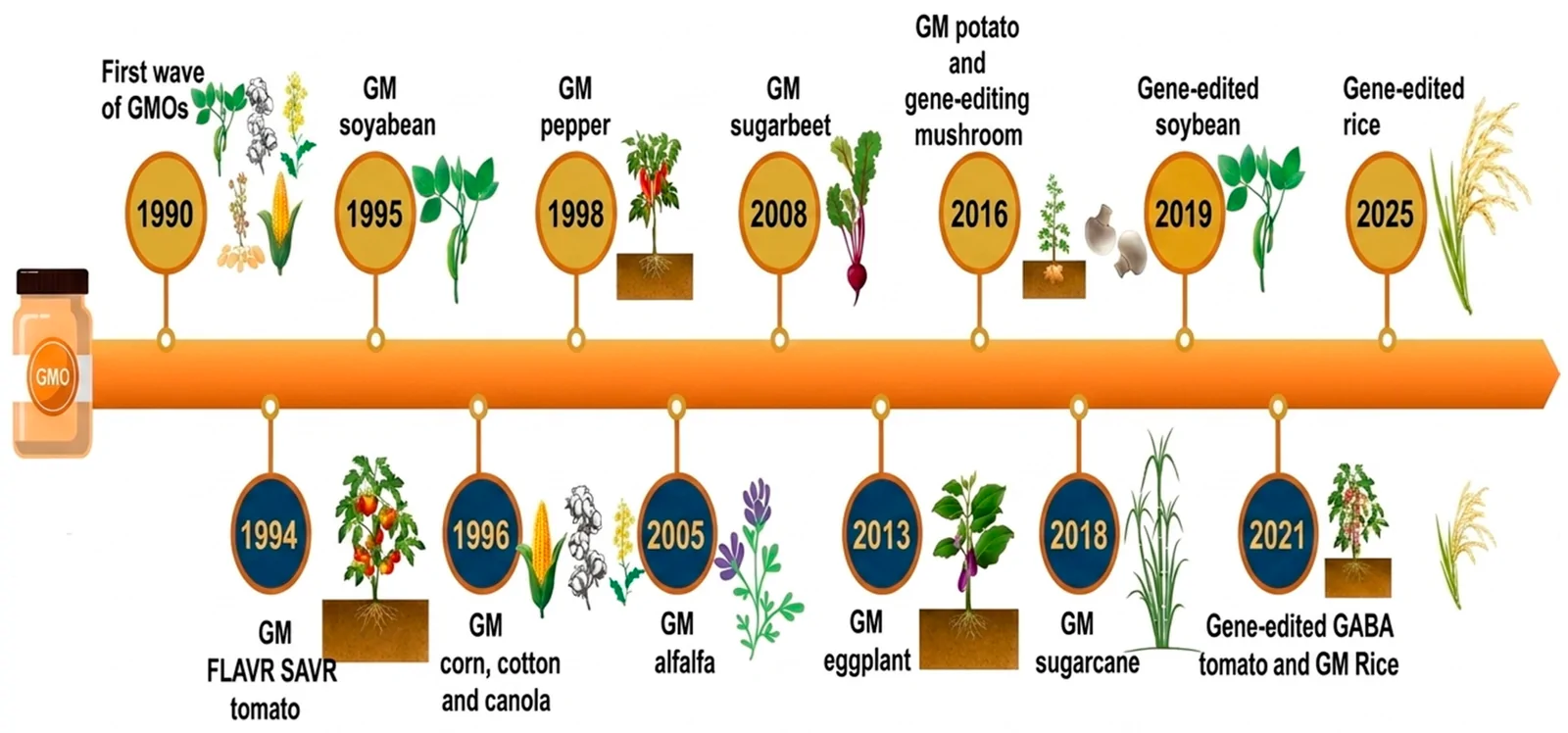
List of the most important GM plants since 1990s (redrawn after trends in plant science).

The first genetically modified plant was an antibiotic-resistant tobacco (1983). In 1994, the US Food and Drug Administration (FDA) approved a transgenic tomato whose ripening is retarded and only ends after harvesting. Subsequently, several GM crops were licensed: rapeseed with a modified oil composition, cotton resistant to the herbicide bromoxynil, Bacillus thuringiensis (Bt) corn, Bt cotton, Bt potatoes, soybeans resistant to the herbicide glyphosate, virus-resistant squash, and several other food, feed or industrial plants (Figure 1).

The list is led by the USA: in 2020, about 92% of corn, 96% of soy, 95% of rapeseed, 96% of cotton, and 99.9% of sugar beet were genetically modified. Ninety-five percent of farm animals consume GM feed, which does not affect their health or their GMO status—which means that their milk and meat is not considered as genetically modified.

#### 4.2.1. Bt Corn

Bt corn (MON 810) is a genetically modified crop (1996) that produces its own natural insect-killing protein from a soil bacterium called *Bacillus thuringiensis*. This built-in protection helps the plant resist harmful pests like the European corn borer, reducing the need for chemical insecticides. These genetically modified plants with Bt protein are grown on a large scale around the world. In the USA, Bt corn acreage grew from approximately 8 percent in 1997 to 87 percent in 2025. Increases in adoption rates for Bt corn may be due to the commercial introduction of new varieties that are resistant to the corn rootworm and the corn earworm (prior to 2003, Bt corn varieties only targeted the European corn borer).

Detractors of Bt corn suggest several challenges, including the potential for effects on non-target organisms and gene flow between Bt corn and non-Bt corn to outweigh any benefits. Other issues to consider include whether insect resistance to Bt toxins can be managed and whether the use of Bt corn is compatible with the other pest control methods. Germany, and later Poland, announced bans on the cultivation of MON 810 on their territory, citing potential harmful effects of the pollen on bees. According to Ricroch et al. (2010), the German decision to ban MON 810 cultivation was “scientifically unjustified,” and no evidence exists to suggest that bees are harmed by the Bt protein [43].

#### 4.2.2. Glyphosate-Tolerant Crops

Glyphosate-resistant crops are genetically engineered plants (like soybeans, corn, cotton, and canola) designed to survive applications of the broad-spectrum Roundup herbicide glyphosate. The first plant was introduced in 1996 (Roundup Ready soybean). A single gene (CP4 EPSPS) derived from a common soil bacterium (*Agrobacterium tumefaciens)* is inserted into the soybean. The bacterial version of the enzyme is naturally tolerant to glyphosate, allowing the soybeans to continue to grow after being sprayed with the herbicide, while other weeds are killed, offering producers a new tool for weed control. The bacterial enzyme is a protein that is easily digested by both humans and animals. In 2025, herbicide-tolerant soybean acreage in the USA reached its highest adoption at 96 percent. GM soybeans offer major agricultural and economic benefits like herbicide tolerance and higher yields, but they also raise concerns regarding “superweeds,” chemical reliance, and strict market regulations. Glyphosate-resistant plants simplify weed control and allow no-till farming, but heavy use has led to resistant “superweeds” [44].

After careful evaluation and assessment, Health Canada has determined that Roundup Ready soybeans are as safe and nutritious as other commercially available soybean varieties and are suitable for food use and accordingly notified Monsanto that it had no objection to their sale.

#### 4.2.3. Golden Rice and Biofortified Crops

Vitamin A deficiency (VAD), which is almost unknown in Europe and North America, is quite common in developing countries of Southeast Asia and Africa because rice, the main food source in these regions, does not contain β-carotene, the precursor of vitamin A. According to the WHO data, this deficiency affects more than 200 million children and women: nearly 14 million children suffer from vision loss, of whom approximately half a million become blind, and 2 million children under five years of age die annually as a result. After eight years of work, Swiss and German researchers produced a GM rice able to synthesize β-carotene in the edible part of the plant. Golden rice was transformed by the addition of two extra genes derived from narcissus and a soil bacterium, increasing β-carotene biosynthesis [45]. The version called “Golden rice 2” contains 23 times more carotenoids, of which β-carotene predominates: one cup of golden rice per day covers 60% of the vitamin A requirement. Since carotenes are hydrophobic, sufficient fat must be present in the diet for golden rice, or any other vitamin A supplement, to alleviate vitamin A deficiency. Golden rice is not patented, and a farmer or subsequent user earning less than $10,000 per year from it need not pay royalties.

In 2018, the USA, Canada, Australia, and New Zealand, and later the Philippines and Bangladesh, allowed its cultivation as food. In 2021, the Philippines became the first country severely affected by VAD to grant final approval for golden rice for cultivation and human consumption, although distribution remains limited. Regulatory hurdles have otherwise stalled its release: approval remains pending in Bangladesh, while legal challenges in the Philippines have at least temporarily halted further research and deployment (see the section on Public Perception and the Acceptance–Rejection Debate below). Leading food agencies of Western nations, including the US FDA, Health Canada, and Food Standards Australia New Zealand, maintain that golden rice fully meets global safety standards; however, because vitamin A deficiency is not a public health emergency in these regions, there is no commercial cultivation there, and it remains strictly a humanitarian export technology. A dozen countries currently participate in the work of the Golden Rice Humanitarian Board. The quarter-century story of Golden Rice has been reviewed by Palmer (2025) [16].

With the process of enrichment called biofortification, it was also possible to produce rice with a high content of folic acid, zinc and iron. Moreover, Australian researchers invented the golden banana by transplanting the gene from the carotene-rich Fei banana into another banana variety. Another example is a GM banana produced by gene editing-induced gene silencing (GEiGS) technology [46]. In this banana, the production of ethylene, which causes rapid browning, is significantly reduced by silencing the polyphenol gene. This fruit has already been approved in the Philippines in 2023.

### 4.3. Genetically Modified Animals

The first genetically modified animal was produced in 1974, and all tissues of the GM mice contained the introduced foreign gene [47]. Some years later, Palmiter and Brinster invented the DNA microinjection, in which the rat growth hormone gene was injected into a mouse embryo—an intervention that produced “giant mice” [48]. This experiment was later repeated with the human growth hormone gene. They also created the first transgenic mouse model for studying human disease, which became an important tool for cancer research. However, during DNA microinjection, the transgene is randomly integrated into the genome (random integration) and expressed in the GM animal. It is also slow, time-consuming and expensive: its efficiency in cattle is only 0.1–0.2%. Newer techniques are easier and more accurate; among them the cloning should be highlighted. Here, the DNA particles precipitated with calcium phosphate are taken up by the cells (transfection). Cells successfully modified with the foreign gene can be selected early with the help of reporter genes. In this way, only the positive cells will be transferred into the target animal cells, so all animals born will be genetically modified. Currently, the most common method of producing GM animals is the cloning of gene-modified cells into the enucleated recipient eggs. This is how the coagulation factor IX-producing GM sheep, which excrete the drug’s raw material in their milk, were born [49].

#### 4.3.1. Increasing Disease Resistance in Livestock

##### PRRS-Resistant Pigs

It is a widespread respiratory and reproductive pig disease, and its estimated damage exceeds 2 billion dollars annually. Research has revealed that the PRRS virus (PRRSV) enters cells by binding to a specific receptor called CD163. Cells with missing or modified receptors cannot be infected by the virus. Therefore, exon 7 of the CD163 gene was deleted by gene editing to create animals that lack functional receptors. Nothing foreign is added to the DNA; the method involves a precise deletion of a portion of a native gene. Because the binding site is removed, the PRRS virus cannot enter the pig’s host cells to cause an infection; these animals became PRRSV-resistant [50].

Thus, from a biological point of view, the procedure was successful; however—due to the ban on genetically modified food-producing animals—PRRS-resistant pigs cannot be introduced into public breeding for the time being. A breakthrough is that in 2025 the FDA had approved its gene-edited pigs after a lengthy review [51].

Recently, Health Canada (2026) approved these pigs, which significantly improve animal welfare, reduce antibiotic use, and combat the serious disease burdening the pork industry [52].

In addition to that, Colombia, Brazil, Dominican Republic and Argentina have issued positive determinations for PRRS-resistant pigs, meaning that those countries have recognized that the pigs are not GMO and should be treated the same as any other pigs.

##### Avian Influenza-Resistant Chickens

Avian influenza is a highly contagious viral disease that, through frequent outbreaks of epidemics, causes substantial economic losses in the poultry industry. Highly pathogenic avian influenza viruses (HPAIVs), particularly those belonging to the H5 and H7 subtypes, can cause severe mortality in poultry flocks and represent a continuous zoonotic threat. Conventional control measures, including vaccination, surveillance, and biosecurity, have limitations in preventing outbreaks. Recent advances in genome editing technologies have enabled the development of poultry with enhanced genetic resistance to avian influenza viruses.

A breakthrough in this field was reported in 2023 by a research group who used CRISPR/Cas9-mediated genome editing to generate chickens with increased resistance to influenza A virus infection. Influenza A viruses depend heavily on host cellular proteins to complete their replication cycle. One of the most important host factors is acidic nuclear phosphoprotein (ANP32A), which serves as a cofactor for the viral RNA-dependent RNA polymerase complex. Previous molecular studies demonstrated that avian influenza polymerases are highly adapted to the avian ANP32A protein and that disruption of this interaction can significantly impair viral replication. The researchers targeted the ANP32A gene, which encodes a host cellular protein required for efficient replication of influenza A viruses. Two amino acid substitutions were introduced into the ANP32A protein, disrupting its interaction with the viral RNA polymerase complex. These modifications were designed to weaken the interaction between the viral polymerase and the host factor without compromising the physiological function of the protein. Challenge studies demonstrated that 90% of the edited chickens remained uninfected following exposure to a conventional challenge dose of avian influenza virus. When exposed to substantially higher viral doses, however, some breakthrough infections occurred [35].

Genetic analysis revealed that viral escape mutants could emerge, enabling partial adaptation to the modified host environment. Further investigations showed that influenza viruses may exploit alternative host proteins, particularly ANP32B and ANP32E, when ANP32A function is impaired. Laboratory studies indicated that simultaneous modification of multiple ANP32 family members could potentially provide stronger and more durable resistance. The avian influenza-resistant chickens developed in this study are generally considered GMO under current European Union regulations, although no foreign DNA from another species was introduced into their genome. The ANP32A locus of CRISPR/Cas9-edited chickens contains targeted modifications within their native genome and do not harbor foreign DNA sequences.

##### Resistance to African Swine Fever (ASF)

African swine fever virus is one of the most serious risks for the world’s pig industry, as nearly 80% of infected domestic pigs die due to an exaggerated immune reaction. This disease is among the most destructive afflictions affecting both domestic and wild pigs. It poses a significant threat to global swine production, with no effective vaccines or antivirals currently available.

African swine fever virus (ASFV) is a large double-stranded DNA virus that poses a significant risk to the pig industry. The high mortality rate can be blamed on a gene called RELA. However, African warthogs and bushpigs survive the same infection without symptoms, even though their RELA gene differs by only a few amino acids from that of domestic pigs. At the Roslin Institute in Scotland, the allele of the RELA gene responsible for the immune response was modified by changing five amino acids. Genetically modified piglets were born, but unfortunately, this GM pig was not yet able to prevent the problem of African swine fever. Later, in an international cooperation, it was possible to identify the protein responsible for the multiplication of the virus inside the infected cells [53]. Their study points to target genes that are candidates for editing to develop pigs resistant to ASF. Although more work is required, this finding represents an important first step towards the generation of ASF-resistant pigs.

Crooke et al. have created gene-edited pigs that are resistant to classical swine fever (CSF), a highly infectious and often fatal disease. Genetic change provided complete protection against infection, with no observable impact on the animals’ health or development. The work proves that precise gene editing can prevent infection by disrupting a pig protein the virus relies on to replicate itself within the cells of the pig. Gene-edited pigs exposed to the virus remained healthy, while unedited animals showed typical signs of disease [54].

However, the prohibition of GM foods would not allow the breeding of ASF-resistant pigs for the time being. Creating a viable, commercial GM pig is largely constrained by regulatory frameworks rather than technical capability. With countries like the US, Japan, and Brazil approving certain gene-edited livestock, researchers are optimistic that approved ASF-resistant pigs could become a viable product within the next 5 to 10 years.

Another effective way of protection is vaccination, which would be allowed at present, but unfortunately an effective and safe ASF vaccine does not yet exist. British researchers, however, using gene editing, developed a vector virus vaccine, by the introduction of eight strategically important ASF virus genes to the place where the viral proteins that activate the immune system are produced [55]. Researchers of the US Department of Agriculture also obtained a live, weakened ASF virus strain by gene editing, which may be suitable to produce an attenuated live virus vaccine [56].

In May 2025, the World Organization for Animal Health (WOAH) adopted its first international standard for ASF vaccines. With this recognition, the global swine health community has formally acknowledged the role that ASF vaccines may play in controlling and mitigating the disease. This is a major step forward, as WOAH standards are the basis for regulations recognized by the World Trade Organization (WTO) for international trade in animals and animal products. These standards directly shape countries’ import and export regulations.

Current ASF vaccines use live attenuated (modified live) strains of the virus and are not inactivated or killed. This means that some virus shedding is expected from vaccinated animals, and adverse side effects are also possible. Countries will need to evaluate these potential adverse effects against the potential benefits of using vaccines. 

Due to virus shedding, the nonspecific clinical signs of ASF, and the absence of a serological test to differentiate vaccinated from infected animals, it may be challenging to define what constitutes an ASF case during vaccine evaluations or implementation. Worldwide tests are in progress, but market-ready, globally authorized vaccines are still in development.

#### 4.3.2. GMOs in Pharmaceutical Production and Industry: Gene Pharming

“Bioreactor” GM animals can produce pharmaceutical raw materials in relatively high concentrations. The homozygous GM lines that can be bred by backcrossing the genetically modified animals will then continuously synthesize the foreign protein for the pharmaceutical industry.

##### Medicines Produced Using GM Technology

Insulin produced by genetically modified bacteria has already been mentioned earlier, but more recently GM cattle that excrete human insulin with their milk have also been created.

The first FDA-approved medicine produced by a GM mammal was an antithrombin formulation. ATryn is an anticoagulant medication containing recombinant human antithrombin. Transgenic goats produce in their milk the raw material of the drug for the treatment of thrombosis, embolism and other blood clotting disorders [57].

A few years later, Ruconest (Conestat alfa), the first recombinant C1 esterase inhibitor, was marketed for the treatment of acute attacks in patients with hereditary angioedema. Genetically modified rabbits produce in their milk the C1 esterase inhibitor missing in patients [58].

Kanuma (sebelipase alfa) is an enzyme replacement therapy developed in 2015, used for treatment of lipase deficiency. Kanuma is approved to treat patients with lysosomal acid lipase deficiency (LAL-D), a rare and life-threatening genetic disorder where harmful fats build up in cells and major organs. The medicine replaces the missing enzyme. For its production, chickens are genetically modified to produce the recombinant form of LAL (rhLAL) in their egg white. After extraction and purification it becomes available as the medication [59].

During the 2014 Ebola epidemic in West Africa, instead of the hyperimmune serum of patients who survived the disease, a mixture of mouse-derived monoclonal antibodies, which were eventually produced by a GM tobacco plant (ZMapp), was successfully used for medical treatment. ZMapp is an experimental biopharmaceutical medication comprising three chimeric monoclonal antibodies.

There are currently licensed vaccines specifically designed to protect against the *Orthoebolavirus zairense* (Zaire ebolavirus), the species responsible for the deadliest Ebola outbreaks. However, no licensed vaccines are currently available for other Ebola species like *Sudan* or *Bundibugyo.* For the time being, there is a serious Ebola outbreak in Congo, Uganda and South Sudan. Using genetic fingerprinting, the illnesses were identified as one of the four types of orthoebolaviruses that cause Ebola disease. Scientists in the University of Oxford, Moderna, and IAVI (a global nonprofit biomedical organization) are fast-tracking new candidate vaccines using mRNA and viral vector platforms.

##### Recombinant Monoclonal Antibody Production in Transgenic Chickens

Transgenic chickens offer a promising platform for recombinant monoclonal antibody (mAb) production. Mukae et al. (2025) investigated the production and downstream purification of recombinant mAb from transgenic chicken eggs [60]. In this system, recombinant IgG accumulated in egg white, providing a non-invasive and scalable production platform. The study focused on overcoming one of the major limitations of egg-based biomanufacturing, namely the efficient separation of recombinant proteins from abundant endogenous egg white proteins such as ovalbumin and ovotransferrin. The purification workflow removed 84.6–93.8% of contaminating egg white proteins while preserving antibody integrity and functionality. The results demonstrated that transgenic chickens could serve not only as biological producers of therapeutic antibodies but also as potentially viable platforms for industrial-scale manufacturing of biopharmaceuticals.

##### Transgenic Cattle as Bioreactors of Human Therapeutic Proteins

Transgenic cattle are genetically modified cows engineered to secrete complex therapeutic proteins—such as antibodies, blood factors, and hormones—directly into their milk. Operating as living “biofactories,” cattle offer massive production capacity and lower costs compared to traditional stainless-steel bioreactors and mammalian cell cultures. Transgenic cattle have long been considered attractive bioreactors for the large-scale production of recombinant therapeutic proteins. The mammary gland is particularly suitable for this purpose because it can synthesize and secrete large quantities of complex glycoproteins while providing post-translational modifications like those occurring in humans. Although major breakthroughs in this field were achieved long ago with the production of human lactoferrin [61], lysozyme [62] and α-lactalbumin [63], current research has focused primarily on refining transgenic technologies and expanding the range of therapeutic molecules produced in bovine milk.

The most significant recent advance was reported by Monzani et al., who successfully generated a transgenic dairy cow capable of producing human proinsulin and insulin in its milk. This is the first documented production of human insulin in bovine milk [64].

The authors constructed a mammary gland-specific expression cassette containing the human proinsulin coding sequence under the control of the bovine β-casein promoter. The transgene was introduced into bovine fibroblasts using a pseudo-lentiviral vector system. Genetically modified fibroblasts were subsequently used for somatic cell nuclear transfer. By transferring the cloned embryos into recipients, one viable transgenic calf was produced. By Western blotting and mass spectrometry both human proinsulin and mature human insulin were detected in the milk. Unexpectedly, the bovine mammary gland appeared capable of processing proinsulin into biologically active insulin through endogenous proteolytic mechanisms. Proteomic analyses further identified that mammary proteases could convert proinsulin to insulin as well as insulin-degrading enzymes. The authors concluded that bovine mammary tissue could function as a highly efficient production system for recombinant insulin and suggested that future transgenic herds could potentially produce insulin at industrial scale.

Although not describing a newly engineered pharmaceutical protein, Yum et al. evaluated the long-term health and genomic stability of transgenic cattle generated through transposon-mediated gene transfer. The authors monitored transgenic cattle over a ten-year period and performed whole-genome resequencing to evaluate structural genomic alterations, somatic mutation rates, and long-term physiological effects. They found no significant increase in mutation burden, copy number variation, structural rearrangements, or health abnormalities compared with conventional cattle [65].

##### Industrial Raw Material: Spider Web Protein

The excellent properties of spider web attracted attention a long time ago: a single gram of the extremely fine thread is 9 km long, and it is stronger than steel and more flexible than rubber. Therefore, it is also suitable for construction, medical, aerospace and military applications. Some ideas from the many uses of spider silk include: biodegradable bottles, flexible bridge suspension cables, biocompatible microsurgical suture materials, production of tear-resistant paper. In addition, thanks to its cell adhesion-proliferation property, spider web protein can control the regeneration of peripheral nerves. It is understandable that several laboratories independently made efforts with genetically modified organisms producing spider web protein. The Canadian Nexia was the first company to produce transgenic goats that secreted 3–5 g/L spider web protein in their milk [66]. From the protein extruded from the aqueous solution, biopolymer fibers of 10–60 µm could be fabricated (spider thread is 2.5–4 µm) [67]. The material was named Biosteel, but due to marketing difficulties, the company was liquidated in 2009. The biologically successful experiment of Nexia was therefore a failure from the utilization point of view. A similar fate came to the American Kraig Biocraft Laboratories, which produced spider web protein in GM silkworms (2010). At the same time, researchers from the Korea Advanced Institute of Science and Technology transferred the gene of the spider Nephila clavipes into *E. coli*. Among the several laboratories, the German AMSilk proved to be the most successful: they transformed protein produced in *E. coli* bacteria into recombinant spider silk, and by exploiting the complete innovation chain, they created marketable products. AMSilk’s Biosteel Fiber is notable for its biodegradability, breaking down in seawater and on land within a few months. As compostable products, they are free of microplastics, with no microplastic washout during the product life. Utilizing a bio-fabrication process that reprograms microorganisms based on spider DNA, the company produces this silk-like material at scale using bacteria and natural fermentation.

In addition, their technology improves the biocompatibility of medical devices and implants by coating them with spider protein because these are “invisible” to the immune system. Recent research shows that AMSilk’s engineered silk implant coatings can prevent infection and reduce post-operative complications of implants. After implantation of coated breast implants, hernia meshes, stents and catheters into the body, the inflammatory reaction and the formation of connective tissue scars are strongly reduced [68,69]. In medical use, recent research shows that AMSilk’s engineered silk implant coatings can prevent infection and reduce post-operative complications of implants.

#### 4.3.3. Food-Producing Genetically Modified Animals

##### Growth-Enhanced Common Carp (*Cyprinus carpio*)

Among genetically modified fish species, growth hormone (GH)-transgenic common carp represents one of the longest-studied and most extensively characterized aquaculture models. Since the late 1980s and early 1990s, several research groups, primarily in China, Israel, and South Korea, have developed transgenic carp carrying either heterologous (human, grass carp, Chinook salmon) GH genes or homologous carp GH constructs under constitutive promoters [70]. The primary objective was to increase growth rate, feed conversion, and overall aquaculture productivity.

Early studies demonstrated that transgenic individuals could exhibit substantially greater body weights than non-transgenic siblings. In some founder lines carrying an autotransgenic carp GH construct driven by a β-actin regulator, certain individuals achieved more than a six-fold increase in body weight during early development and reached approximately 5 kg within 8–9 months, representing up to a ten-fold increase relative to non-transgenic controls [71]. However, growth enhancement was not uniform among all transgenic lines. Studies comparing different integration events showed considerable variation in growth response, indicating that the genomic insertion site, transgene copy number, and genetic background strongly influence the phenotype. Some transgenic lines exhibited significant growth acceleration, whereas others showed little or no improvement compared with controls [72].

Recent studies have demonstrated that growth enhancement is accompanied by extensive metabolic remodeling. GH-transgenic carp display lower lipid accumulation in serum, liver, and whole-body tissues [73]. Enhanced hepatic lipolysis and increased mitochondrial β-oxidation capacity were observed, resulting in more efficient lipid utilization. Specific growth rate (SGR) was approximately 12% higher and feed efficiency about 17% higher than in wild-type fish under comparable feeding conditions. Recent studies have demonstrated that growth enhancement is accompanied by extensive metabolic remodeling.

Food safety evaluations conducted in China reportedly found no pathological abnormalities associated with consumption of GH-transgenic carp, although commercial approval has remained limited due to regulatory and environmental concerns. Despite promising production traits, biosafety and ecological concerns have limited widespread commercialization.

##### Fast-Growing GM Salmon

Supplying the growing population with proteins of animal origin is an increasing challenge. The feed conversion of animal species differs: to produce 1 kg of live weight, cattle require 8, pigs 3, poultry 2 and fish only 1.2 kg of feed. It is therefore understandable that fish consumption plays an important role; it has increased by 6.6% per year in the last half century. However, due to the overfishing of the seas and inadequate fish management, the only alternative is fish farming. According to an FAO estimate, the current 50% share of aquaculture will rise to over 60% by 2030. Among the fish species, salmon is extremely important, as they are rich in omega-3 fatty acids and very efficient protein producers. The two largest exporters of Atlantic salmon fished from natural waters are Norway and Chile, which means that the price of the product is increased by considerable transportation costs, and thus also a significant ecological footprint. In the case of farmed salmon, however, it takes three years to develop fish of market size and weight.

Researchers from a Canadian company inserted the gene of interest from the continuously developing chinook salmon into the genome of the Atlantic salmon, which does not grow in winter. Integration and expression of the chinook salmon gene are regulated by a promoter sequence from the Ocean pout (*Zoarces americanus*) [74]. Due to its higher growth rate, genetically modified Aquadvantage salmon needs 25% less feed than “wild” salmon to reach market weight. The GM fish are disease- and antibiotic-free because they are reared in closed tanks located on land under optimal conditions. GM fish stock will never be released into open waters.

The production of GM salmon begins with the selection of wild salmon individuals with the best genetics. The foreign chinook salmon gene is introduced into and carried by the male line, and their sperm is used to fertilize the eggs of the non-transgenic Atlantic salmon. After fertilization these eggs are pressure-sterilized; thus the fish stock hatching from them consists exclusively of infertile females. In this way, even in the worst-case scenario, wild stocks are saved from interbreeding in any case. The hatched salmons are then transported to closed nurseries located on land, where they grow until the market size. The incoming and outgoing water flowing into and out of the tanks is under complete control; 95% of the waste is filtered out, and the treated wastewater can be used for other purposes.

GM salmon experiments began in 1989, and the first transgenic salmon was ready within three years, but the subsequent approval process took three decades. Finally, in 2015, the FDA concluded that the fish are safe for humans and animals to eat, that the rDNA construct is safe for the animal, and that the claim of faster growth is confirmed; AquAdvantage salmon thereby became the first GM animal approved for human consumption.

##### Environment-Friendly GM Pigs

Phosphorus is necessary for pig development and reproduction, but 50–70% of the phosphorus in feed is bound to phytic acid, which pigs cannot digest due to the lack of a decomposing enzyme (phytase), resulting in reduced development and mineral (Ca, P, Fe) deficiency. The phytase enzyme given as a feed supplement could make phosphorus digestible by cleaving it from the phytin bond, but it is expensive.

Canadian researchers approached the problem differently: they put phytase gene from *E. coli* together with a regulatory sequence obtained from a mouse. By microinjecting this gene construct, a GM pig (Enviropig) was created that produces a phytase enzyme with its saliva, making dietary phosphorus digestible and reducing or eliminating the need for phosphorus supplementation. Enviropigs release 65% less phosphorus in excrement, considerably reducing the environmental burden associated with phosphorus runoff and downstream eutrophication [75,76].

Enviropig was the first GM animal produced for environmental protection but has not been approved, as society was still uncertain about GM animals. Based on the available evidence, there was no specific governmental regulation or legal ban that terminated the Enviropig program. Instead, the project ended because its principal industry sponsor withdrew financial support, after which the University of Guelph discontinued the live breeding program and euthanized the remaining animals [77]. GM animals have been eradicated, but the genetic material is preserved in a repository. Regulatory approval processes remained formally open, but commercialization never occurred due to lack of industry adoption and limited market acceptance.

A few years later, a similar GM pig was produced in China [78] that has less impact on the environment and grows faster, but due to current legislation, even this cannot be introduced into public breeding today.

It is promising that alpha-galactose-free knockout pigs (GalSafe) received FDA approval in 2020 to be consumed as food by allergic patients with alpha-gal syndrome. Since the same sugar is responsible for the hyperacute rejection following the implantation of pig organs (see the next chapter), the gene knockout pig can also be used for therapeutic purposes (xenotransplantation).

#### 4.3.4. Organ Donor GM Pigs for Xenotransplantation

The idea of organ transplantation dates to the early Middle Ages when, according to oral tradition, St. Cosmas and St. Damien transplanted a Moor’s leg to replace the ulcerated leg of a white patient. In fact, organ transplantation began with a cornea transplant in the early 20th century, followed by a kidney transplant half a century later. The real breakthrough was the first heart transplant performed almost 60 years ago [79] which was followed by the successful transplantation of more and more organs and tissues, and transplantation medicine developed into a separate discipline. The ever-increasing discrepancy between the number of organs available for transplantation and the number of patients in need is a concern, as the number of transplants has more than doubled, while the waiting list has grown nearly six-fold. Replacement or support of the diseased organs by machines (artificial kidney, artificial heart and lung) is only a temporary help because their performance, lifespan and especially their size are not even comparable to the original organs. With traditional means (searching for donors, better organization of transport), the problem of opening gaps cannot be solved; because of this, thousands of patients on the waiting list must die. In the USA, there are >90,000 people waiting for only 25,000 kidneys. Most of them (≥80%) must wait up to five years, which many of them are no longer able to afford [80]. Xenotransplantation—the use of organs from a foreign species—has focused on baboons and, more prominently, pigs, owing to their accessibility, reproductive speed, low zoonotic risk, and physiological similarity of organ size to humans. Heart valves, skin, and corneas have already been transplanted from pigs into humans. However, kidneys, hearts and other visceral organs have so far only been implanted in non-human primates, as the implanted foreign organs (xenografts) are immediately rejected due to incompatibility. The first of the four rejection phases (hyperacute rejection) manifests itself in the fact that right after connecting the vascular network, the implanted organ turns blue and is rejected, for which the α-galactose sugar chains are responsible. The other three rejection phases are slower and can be controlled by immunosuppressive drugs. In order to prevent hyperacute rejection, it was necessary to create pigs in which the α-1,3-galactosyltransferase gene had been knocked out: several research groups succeeded in producing such pigs. These are potential organ donors whose organs are not rejected by hyperacute rejection, since the sugar that triggers an immediate human immune reaction is missing from the surface of their cells. The α-1,3-galactosyltransferase gene was successfully inactivated in 2003. In 2017, 62 porcine endogenous retroviruses were inactivated, and twenty additional genes were modified whose gene products cause an immune reaction and the formation of blood clots. During the production of “humanized pig donors”, the gene pool was modified at a total of 69 points, including the elimination of glycan antigens, the implantation of some human genes, and the inactivation of endogenous porcine retroviruses [38].

Nowadays, xenografts have reached the clinical phase: in 2021, the kidney of a gene-edited pig was connected to the machine-maintained circulation of a brain-dead man [81]. In the organ donor animal, four pig genes were knocked out, and six human genes were inserted into the genome, which helps the human body accept the transplanted organ. After transplantation of the organ, no hyperacute rejection was observed; the transplanted kidney filtered and secreted urine for two days. In March 2024, a living (62-year-old) patient received a gene-edited pig kidney [82]. Following this, U.S. clinical trials gained FDA approval, resulting in multiple patients safely receiving gene-edited pig kidneys.

This operation was a milestone in xenotransplantation, as 80% of the nearly two hundred thousand names on the waiting list are made up of those waiting for a kidney.

GM pig kidney recipients have shown promising, albeit varied, survival rates in recent clinical trials, with a record survival of approximately nine months achieved by a 67-year-old patient. Other human recipients have lived for roughly 60 to 130 days. Recently a gene-edited porcine kidney functioned in a human for a record-breaking 271 days before being removed due to continuous proteinuria [83].

In 2022, a gene-edited pig heart was transplanted, under a case-by-case permit, into a terminally ill 57-year-old man; his death two months later may have involved several factors, including possible transmission of cytomegalovirus from the donor pig despite negative preliminary testing [84]. A second pig heart transplantation the following year resulted in survival of one and a half months before death from rejection [85]. To date, two living patients have received genetically modified pig heart transplants, both with terminal heart disease and ineligible for human transplants; there are currently no authorized clinical trials of pig heart transplantation in living humans, and despite progress in overcoming hyperacute rejection, adaptive cellular and humoral immunological barriers remain [86,87]. In 2025, a landmark procedure in China attached a liver from a six-gene-edited Bama miniature pig to a brain-dead human patient; the organ produced bile and showed no signs of immediate rejection [83]. Researchers have identified more sensitive viral screening and improved anti-rejection treatments as necessary for future clinical success [88].

## 5. Regulatory Landscape

### 5.1. Global Regulatory Status and Label Laws

The legal and regulatory status of GM foods varies considerably by country, with some nations banning or restricting them and others permitting them under widely differing degrees of regulation. One point of debate is labeling: it is voluntary in the USA and Canada but mandatory in Europe for feeds and foods containing more than 0.9% GM ingredients. More than 60 countries regulate genetically engineered crops, distinguishing between total bans, farming prohibitions, and mandatory labeling requirements.

Only a few nations completely outlaw both growing and importing GMOs, including Algeria, Benin, Bhutan, Madagascar, Peru, Russia, Serbia, Venezuela, and Zambia. Many other nations—including France, Germany, Austria, Italy, Hungary, Greece, Turkey, Saudi Arabia, and Switzerland—prohibit farmers from planting GMO crops locally to protect native biodiversity while still permitting imported GMO goods for food or animal feed. The EU applies rigorous, case-by-case scientific risk evaluations through the European Food Safety Authority (EFSA); while several GM lines are approved for import, mainly for feed, individual member states retain the legal right to ban cultivation on their own soil.

Among countries permitting GMO ingredients, mandatory labeling laws take two general forms. Process-based systems, used in Brazil, China, and the EU, require labeling whenever any ingredient is derived from a GM source, even when no modified DNA or protein remains detectable in the final processed product. Product-based systems, used in Australia, Japan, and New Zealand, require labeling only when novel GM material or protein is present in the final consumable food. The United States and Canada regulate biotech crops based on “substantial equivalence” to conventional crops, mandating disclosure (text, symbol, or digital/QR link) for certain bioengineered foods while exempting highly refined derivatives and animal-fed meat or dairy. Voluntary “GM-free” labels are also permitted in markets such as the EU for producers wishing to market products as free of biotechnology, provided the claims remain transparent and factual.

A recent survey of the 196 United Nations member states summarized their regulatory status regarding transgenesis: 24 countries allow it for any use, one allows it for import only, 37 have legislation under discussion, three do not allow it, seven regulate it specifically as transgenics, 114 have no legislation, and 10 have no data available. Countries that have authorized transgenic plants are 22.6% more likely to also approve gene-edited plants than those that have not [9]. In the EU specifically, in vitro mutagenesis carried out on plant cells was recently removed from the GMO directive, and the European Commission has authorized genetically modified corn as food and feed and renewed the approval of two GM rapeseed varieties, both judged by EFSA to be as safe as their conventional counterparts [8,9].

### 5.2. New EU Legislation on New Genomic Techniques

The EU has adapted its legislation on plants produced with new gene editing techniques, termed “new genomic techniques” (NGTs) [89]. The new law was adopted by the European Parliament on 17 June 2026 and will apply from mid-2028, marking a shift toward regulation based on the genetic characteristics of the final plant rather than the method used to produce it. Without the new rules, all NGT-edited plants would fall under the existing GMO rules, written before modern gene editing techniques were invented and among the strictest in the world—meaning that even a single-letter DNA edit, of the kind that regularly occurs spontaneously in nature, would face the same authorization requirements as a complex edit such as inserting a bacterial gene into corn.

Gene-edited plants are now divided into two categories. NGT-1 plants carry a limited number of changes that could have occurred through conventional breeding and are treated as conventional plants; plants engineered for herbicide tolerance or to produce insecticidal substances cannot be classified as NGT-1. NGT-2 plants, which have undergone more extensive or complex genetic modification, remain subject to the existing strict GMO rules, including risk assessment, authorization, labeling, traceability, and member-state opt-outs for cultivation. Neither category is permitted in organic production. Currently, the only GM crop approved for cultivation in the EU is MON810, an insect-resistant corn grown mainly in Spain for animal feed, while several other GMOs are authorized for import; all require a case-by-case scientific risk assessment by EFSA together with strict labelling and traceability rules.

## 6. Public Perception and the Acceptance–Rejection Debate

### 6.1. Scientific Consensus and Public Opinion

**Figure 2:**
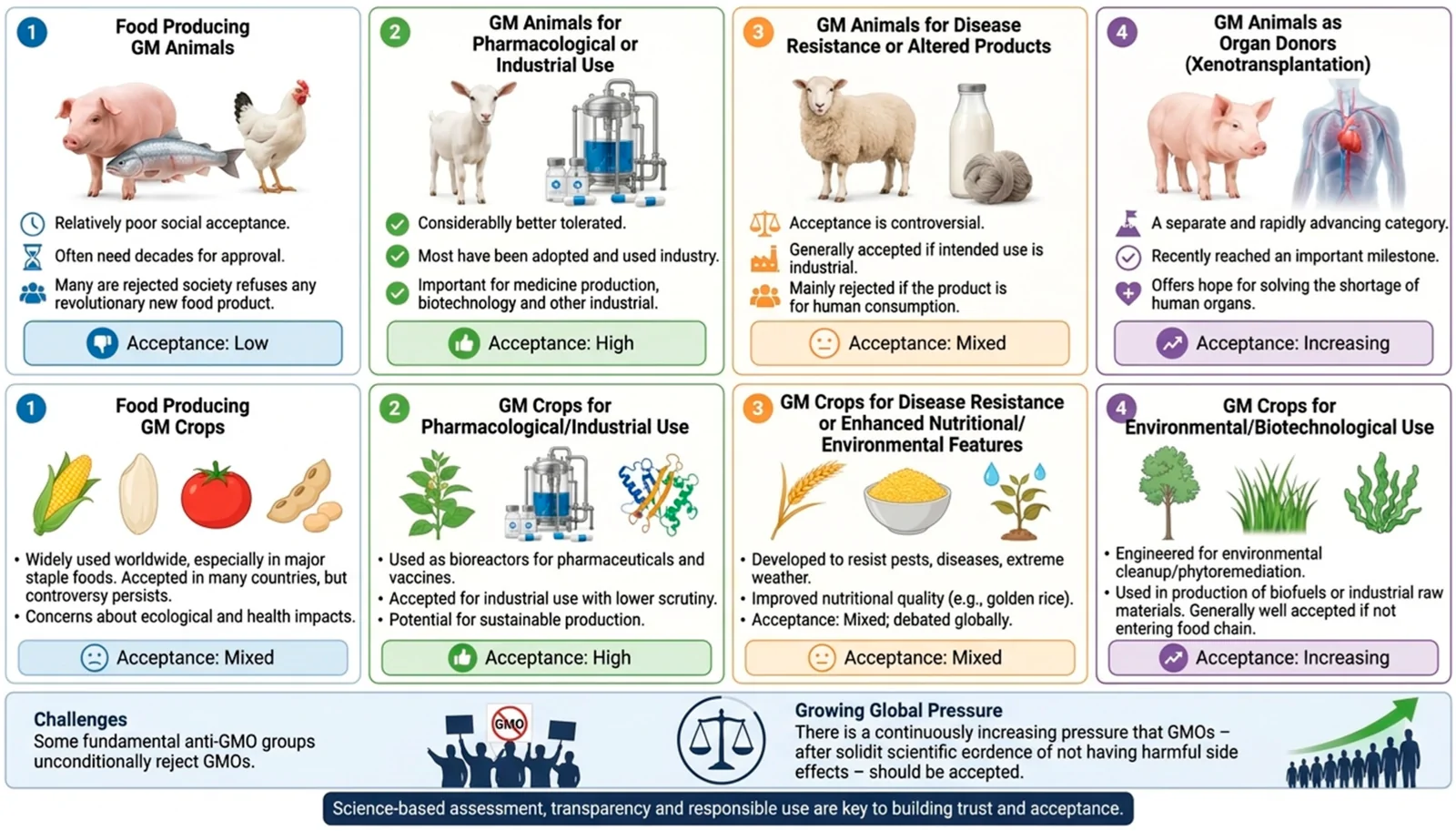
Acceptance of GMOs according to their intended use.

Public perception of GMOs is controversial (Figure 2). Products and pharmaceutical raw materials synthesized by GM organisms for industrial processing are generally well supported by the public. Social acceptance of research about gene therapy and xenotransplantation is also favorable.

However, GM food products are mostly rejected, although so far there is no evidence indicating any harmful effects of genetically modified foods on the human population. There is a scientific consensus that currently available food derived from GM crops poses no greater risk to human health than conventional food, but that each GM food needs to be tested on a case-by-case basis before introduction [90]. There is no evidence for the idea that the consumption of approved GM food has a detrimental effect on human health [91]. Some scientists and advocacy groups, such as Greenpeace and World Wildlife Fund, have nonetheless called for additional and more rigorous testing of GM food [92]. Despite long-standing, widespread consumption of GM soybean, of which 90% is produced this way, public perception of GMOs for food purposes varies considerably, and members of the public remain much less likely than scientists to perceive GM foods as safe [93].

The most intensely debated GM plants globally include corn, soybean, cotton, golden rice, and Bt eggplant (Brinjal). These crops spark fierce arguments over corporate seed monopolies, environmental impacts on non-target insects, chemical herbicide resistance, and the ethics of altering staple global food supplies. Recent consumer studies, however, show increased acceptance of gene editing compared with traditional transgenic GMOs. Regulatory divergence has established new paradigms differentiating precision breeding from conventional genetic modification, and gene-edited crops have attained GMO-free status in several jurisdictions, circumventing traditional regulatory obstacles; the first generation of CRISPR crops has now entered commercial markets, demonstrating practical viability and consumer acceptance [94].

### 6.2. Golden Rice as a Case Study in the Acceptance Debate

Golden rice illustrates how public and organizational opposition can shape the trajectory of a genetically modified product independent of its safety profile. Despite being freely licensed and endorsed by regulatory agencies in multiple countries, golden rice has faced considerable opposition from environmental and anti-globalization groups, which have campaigned against what they describe as dangerous “frankenfoods” and, in some cases, destroyed experimental plots. Mark Lynas, a former anti-GMO activist, has publicly criticized environmental organizations, including Greenpeace and organic trade groups such as the UK Soil Association, for what he characterizes as disregard of scientific evidence on genetically modified crop safety and benefits in favor of ideological positions [95]. In 2016, 133 Nobel Prize laureates called on Greenpeace to end its campaign against GMOs, particularly golden rice.

Approval has nonetheless proceeded unevenly: the Philippines granted final approval for cultivation and human consumption in 2021, but its Court of Appeals issued a cease-and-desist order in 2024 following a petition from opposing farmers and scientists, a decision that critics argue puts children’s lives at risk. As a result, 44 years after the experiments began and 26 years after the first reported success [96], golden rice remains at the center of debate, despite an estimated 266,000 annual deaths attributable to vitamin A deficiency [97]. This case illustrates a recurring pattern in the GMO debate: regulatory and scientific endorsement does not, by itself, determine societal or political acceptance.

### 6.3. Weighing Potential Risks and Benefits

Discussions of GMO acceptance and rejection commonly center on a comparison of potential benefits and risks across several dimensions, including human health, food security, animal health and welfare, the environment, evolution, biodiversity, medicine, and agriculture. Table 3 summarizes the representative potential benefits and risks associated with GMOs across these dimensions, along with the key questions typically used to guide their assessment.

Summarized concisely, arguments commonly raised in favor of genetic modification include that GMO crops have been shown to be safe through extensive testing and use, and can even increase the safety of common foods; that genetic modification can improve crop yields, lower food prices, and increase nutritional content, helping to address world hunger; and that growing GMO crops can bring environmental benefits, such as reduced pesticide use, less water waste, and lower carbon emissions. Arguments commonly raised against genetic modification include that GMO crops have not been proven safe for long-term consumption through human clinical trials; that altering the genetic makeup of plants could introduce toxins or trigger allergic reactions; and that GMO crops can harm the environment through increased use of toxic herbicides or pesticides [98].

### 6.4. Overall Discussion

Genetically modified and gene-edited organisms have emerged as important tools for addressing challenges in agriculture, food production, animal health, biotechnology, and human medicine. Their potential benefits include improved nutritional quality, enhanced resistance to pests and diseases, increased production efficiency, reduced dependence on some agricultural inputs, production of pharmaceuticals, and the development of animals with potential applications in xenotransplantation. From a human health perspective, current scientific and regulatory assessments indicate that approved GM foods are not inherently more hazardous than conventionally produced foods when evaluated on a case-by-case basis, while some applications may provide direct or indirect health benefits, such as nutritional biofortification or the production of therapeutic proteins. Nevertheless, genetic modification is not without potential drawbacks or uncertainties. Depending on the organism and intended application, concerns may include allergenicity, toxicity, unintended genetic or phenotypic changes, altered nutritional composition, environmental effects, animal welfare issues, and uncertainties associated with long-term or large-scale deployment. Importantly, these potential risks cannot be generalized to all genetically modified organisms because the biological characteristics and consequences of individual modifications differ substantially. The continuing controversy surrounding GMOs therefore reflects not only questions of biological safety, but also ethical, environmental, socioeconomic, regulatory, and cultural considerations. Overall, the available evidence supports a balanced, scientifically grounded and case-specific assessment of each application rather than a uniform classification of GM organisms as either beneficial or harmful.

## 7. Conclusions

The overarching scientific consensus indicates that genetic modification is neither inherently harmful nor fundamentally risk-free; rather, it represents a powerful biological instrument whose safety, efficacy, and utility are strictly contingent upon context. The socioeconomic and ecological implications of GMOs depend entirely on the specific organism modified, the nature of genetic alteration, the resulting phenotypic traits, and the environmental framework of their deployment. Consequently, evaluating biotechnological innovations requires a rigorous, case-by-case assessment that balances empirical scientific evidence, projected benefits, and potential adversarial risks, rather than treating GMOs as a single, uniform category.

Globally, public and market acceptance of GMOs remains deeply fragmented, shaped by a complex interplay of geographical variations, application types, institutional trust, and scientific literacy. While agricultural biotechnology has seen rapid adoption by producers, consumer attitudes have evolved from the polarized debates of the 1990s into a more nuanced discourse driven by contemporary precision gene editing tools like CRISPR. Societal comfort is significantly higher for biomedical applications—such as biopharmaceuticals, gene therapies, and xenotransplantation research—and humanitarian biofortified crops, compared to commercial transgenics or broad-scale agricultural animal modifications. Furthermore, a pronounced knowledge gap persists, wherein higher technical literacy correlates with risk tolerance, while low familiarity frequently manifests as generalized risk aversion.

Geopolitical and Regional Disparities: The regulatory and ideological frameworks governing biotechnological deployment remain starkly divided across specific global regions. In North America, a robust public and market consensus exists, prioritizing crop biotechnology as a critical mechanism for food security and economic affordability, though debates on mandatory labeling persist. Conversely, the political and social environment in Europe remains largely hostile or deeply cautious toward cultivating GM crops and introducing transgenic animals; this stance is backed by strict precautionary regulatory frameworks and continuous consumer demand for non-GMO alternatives. Meanwhile, developing nations in Africa and Asia experience highly complex socio-political dilemmas, where the immense potential benefits of biotechnology for localized food security directly clash with deep-seated anxieties over corporate seed monopolies, technological dependency, and the global control of agricultural infrastructure.

The Risk–Benefit Matrix and Future Governance: The dichotomy between potential bio-advancements and multifaceted risks underscores the necessity for transparent governance. While proponents emphasize enhanced crop yields, reduced chemical pesticide dependencies, climate resilience, and optimized animal welfare through disease resistance, critical concerns persist regarding unintended long-term health effects, toxicity, allergenicity, and altered nutritional compositions. A pivotal distinction must be maintained between controlled, individual-level laboratory containments and population-level environmental releases. The deliberate introduction of modified organisms into the wild introduces irreversible ecological threats, including horizontal gene flow, disrupted ecological relationships, evolutionary anomalies, and aggressive competition with wild species. Ultimately, public skepticism intensifies when multinational corporations are perceived as the primary beneficiaries over small-scale farmers or consumers. Moving forward, genetic biotechnology must not be viewed as either a universal remedy or as an existential threat, but as a sophisticated biological tool requiring responsible development, stringent monitoring, and profound ethical oversight.

## Figures and Tables

**Table 1:** Some important milestones of gene modifications.

Era	Year(s)	Milestone/Description
Early Foundations and Recombinant DNA Introduction and Early Skepticism 1953–1980s	1953	Watson and Crick discover the double-helix structure of DNA
	1973	Cohen and Boyer created the first recombinant DNA organism by inserting foreign genes into *E. coli* bacteria
	1982	FDA approves synthetic human insulin (Humulin) produced by bacteria as the first commercial recombinant DNA drug
	1986	First field tests of genetically engineered plants (tobacco and tomatoes)
Commercial GMO Era Polarization and transparency demands 1990s–2000s	1994	The Flavr Savr tomato becomes the first commercially grown GM food allowed in U.S.
	1995/96	Insect-resistant Bt corn and herbicide-tolerant Roundup Ready soybeans gain regulatory approval and commercial planting
	2003	The WHO and FAO establish international safety standards for evaluating GMO foods
The Gene Editing Revolution Softening resistance 2010s–Present	2012	Doudna, Charpentier and colleagues publish a precise, programmable gene editing tool (CRISPR-Cas9)
	2015	The FDA approves the first genetically engineered animal for human consumption (AquAdvantage salmon)
	2016	U.S. legislation introduces mandatory “bioengineered” food labeling rules
	2016	Invention of double-strand break-free base editing
	2019	The FDA completes safety consultations for the first food derived from a genome-edited plant (high-oleic soybean)
	2019	Introduction of prime editing (no double-strand break)
	2020	The FDA approved a genetically modified pig line (GalSafe pigs) engineered to eliminate alpha-gal sugars, making meat safe for people with meat allergies
	2021	A CRISPR-edited tomato containing high GABA level (for blood pressure) went on sale, marking a commercial milestone

**Table 2:** Representative genetically modified (GM) and gene-edited plants and animals, their intended traits, potential implications for human health, evidence, regulatory status, and representative references.

Organism	Technology	Genetic Modification/Edited Target	Intended Trait	Potential Human Health Benefits	Evidence for Human Health Effects	Potential Risks or Concerns	Regulatory Status *	Representative References
Bt maize	Expression of *Bacillus thuringiensis* (Bt) Cry proteins for insect resistance	*cry1Ab*, *cry1Ac*, *cry3Bb1*	Insect resistance	Reduced mycotoxin contamination due to lower insect damage; improved food safety.	Strong evidence. Lower fumonisin and aflatoxin levels consistently reported. No evidence of adverse health effects from approved events.	Public concerns regarding allergenicity and long-term safety have not been supported by regulatory assessments.	Approved in several countries (USA, Canada, Brazil, Argentina, South Africa, China, EU imports).	Nicolia et al. (2014) [14] Li et al. (2026) [15]
Golden rice	Transgenic (GM)	*psy* and *crtl* genes	β-carotene production	Improved vitamin A intake in populations at risk of deficiency.	Moderate to strong evidence. Human intervention studies demonstrate effective conversion of β-carotene into vitamin A.	Limited public acceptance. Nutritional impact depends on adoption and dietary habits.	Approved in Australia, New Zealand, Canada, and the USA (Food, import).	Palmer (2025) [16]
High-oleic soybean	Gene editing (TALEN/ CRISPR/Cas9)	*FAD2-1A* *FAD2-1B*	Increased oleic acid	Improved nutritional profile of edible oils.	Strong evidence for altered fatty acid composition.	No additional food safety concerns identified.	Approved in USA, Canada, Brazil, Argentina, Japan, Australia, New Zealand. Import: China, EU.	Fu et al. (2022) [17] Da Rosa et al. (2025) [18]
Arctic^®^ apple	Agrobacterium-mediated transformation & RNA interference for PTGS	Knockdown of polyphenol oxidase (*PPO*) gene family (*PPO2*, *GPO3*, *APO5*, *pSR7*)	Suppression of polyphenol oxidase	Reduced enzymatic browning, leading to less food waste and improved product quality.	Strong evidence for compositional equivalence to conventional apples.	No additional health risks identified during regulatory assessment.	Approved in USA and Canada (no active commercial planting yet).	Klocko (2025) [19]
AquAdvantage^®^ salmon	Transgenic (GM)	Chinook salmon growth hormone gene	Accelerated growth	Improved availability of nutritious seafood rich in omega-3 fatty acids.	Strong evidence. FDA concluded food was safe. Nutritional composition equivalent to conventional Atlantic salmon.	Escape, competition and gene flow into wild salmon populations. Environmental risks require biological containment and case-specific assessment.	Legally approved in USA and Canada. Production completely ceased as of late 2024/2025 due to commercial unviability.	Clifford (2014) [20] Zheng et al. (2023) [21]
GalSafe pig	Gene editing (knockout)	*GGTA1*	Elimination of α-Gal epitope	Meat potentially suitable for individuals with α-Gal syndrome; source of safer xenotransplantation materials.	Moderate evidence. α-Gal elimination confirmed; clinical utility under active investigation.	Limited clinical experience; ethical considerations.	FDA-approved (USA).	Stelcer et al. [22]. (2025)
PRRS-resistant pig	CRISPR/ Cas9	Removes scavenger receptor cysteine-rich domain 5 (SRCR5) of the CD163 protein	Resistance to PRRS virus	Reduced need for antimicrobial treatments; healthier livestock; potential improvement in food security.	Moderate to strong evidence in experimental and commercial breeding programs.	Potential for viral adaptation/ mutational escape bypassing the CD163 pathway; international trade disruptions due to asynchronous global approvals.	Approved in USA, Canada. Granted non-GMO/conventional status in Argentina, Brazil, Colombia, Uruguay, and Dominican Republic.	Burkard et al. (2018) [23] Lusk (2025) [24]
Gene-edited hornless (polled) cattle	CRISPR/ Cas9	*POLLED* allele introduction	Naturally hornless cattle	Improved animal welfare by eliminating dehorning; indirect public health benefit through improved livestock management.	Limited direct human health evidence.	Unintended integration of bacterial plasmid donor DNA and antibiotic-resistance marker genes into the host genome; unknown long-term pleiotropic effects of the POLLED locus.	USA: Regulated under “new animal drug” protocols on a case-by-case “enforcement discretion” framework. Brazil/Argentina: Classified as conventional/non-GMO.	Grobler et al. (2021) [25] Van Eenennaam (2019) [26]
Heat-tolerant cattle	CRISPR/Cas9	*SLICK* allele	Enhanced heat tolerance	Improved sustainability of livestock production under heat stress; supporting food security.	Limited direct human health evidence.	Potential unintended pleiotropic effects of PRLR gene truncation; increased susceptibility to seasonal cold stress.	USA: Cleared for food use. Brazil/Argentina: Formally deregulated and classified as non-GMO/conventional.	Porto-Neto et al. (2018) [27] Pozzebon et al. (2024) [28]
MSTN-edited cattle	CRISPR/Cas9	*MSTN*	Increased muscle growth	Greater lean meat production and production efficiency.	No direct clinical evidence, demonstrating either a health benefit or a health hazard in humans consuming meat from MSTN-edited cattle.	Unintended genomic alterations, compositional changes and allergenicity/toxicity concerns requiring product-specific assessment. Animal welfare concerns associated with hypermuscularity (dystocia, locomotor problems, heat stress and altered organ proportions).	Brazil: Specific positive regulatory classification exists for MSTN-edited Nelore bull semen.	Gim et al. (2022) [29] Santomassimo et al. (2026) [30]
Atryn-producing GM goat	Transgenic animal technology; mammary-gland bioreactor for recombinant protein production	Human antithrombin (AT) cDNA expressed under a goat β-casein promoter, resulting in secretion of recombinant human AT (ATα; ATryn) in milk	High-level production of recombinant human AT for pharmaceutical use	Availability of a recombinant, non-plasma-derived source of antithrombin for patients with hereditary antithrombin deficiency.	Strong clinical and regulatory evidence supports the intended therapeutic use of the recombinant protein, although the evidence concerns ATryn as a pharmaceutical rather than beneficial effects of the GM goat itself.	Host-specific post-translational modification (glycosylation). Hypersensitivity to goat or goat-milk proteins. Excessive AT activity may increase the risk of bleeding. Transgenic livestock require controlled breeding, containment, genetic stability monitoring and rigorous purification and characterization of the recombinant product.	USA: Approved by FDA (2009) for prevention of peri-operative and peri-partum thromboembolic events in hereditary antithrombin deficiency.	Zhou et al. (2005) [31] Adiguzel et al. (2009) [32] Kling (2009) [33] Gavin (2014) [34]
Avian influenza-resistant chicken	CRISPR/Cas9	*ANP32A* gene editing	Reduced susceptibility to avian influenza virus	Mitigating zoonotic and pandemic risks; reduction in human exposure; safeguarding of global food security and public nutritional health.	Emerging evidence from experimental studies.	Viral adaptation; potential selection for viral variants with enhanced zoonotic potential or increased replication efficiency in humans.	USA: Approval pending. UK: Biosafety review	Idoko-Akoh et al. (2023) [35]
Gene-edited pigs for xenotransplantation	CRISPR/Cas9-based multiplex genome editing, often combined with somatic cell nuclear transfer	Triple knockout of *GGTA1*, *CMAH*, *B4GALNT2*; inactivation of PERV; insertion of human genes such as *CD46*/*CD55*, *THBD*, *EPCR*, *CD47* and other immune/coagulation regulators	Production of safer donor organs	Potentially provides an alternative and scalable source of organs (kidneys, heart).	Strong preclinical evidence in non-human primates. Clinical proof-of-concept has been achieved: gene-edited pig kidneys have functioned in living human recipients.	Antibody-mediated and cellular rejection, thrombotic microangiopathy, inflammatory injury, incompatibility of coagulation pathways, PERV and other zoonotic infections, graft overgrowth/ function, and the need for intensive immuno-suppression. Long-term safety and durability in humans remain uncertain.	Experimental/ clinical trial stage; not an established routine clinical therapy.	Lutz et al. (2013) [36] Niu et al.(2017) [37] Anand et al. (2023) [38] Anderson et al. (2024) [39] Alobaidi (2025) [40]

* Regulatory status reflects current approvals or development status and varies between jurisdictions. Abbreviations: AT: antithrombin; CRISPR: Clustered Regularly Interspaced Short Palindromic Repeats; FDA: Food and Drug Administration; GM: genetically modified; MSTN: Myostatin; PERV: porcine endogenous retrovirus; PRLR: Prolactin Receptor; PRRS: Porcine Reproductive and Respiratory Syndrome; PTGS: Post-Transcriptional Gene Silencing; TALEN: Transcription Activator-Like Effector Nuclease.

**Table 3:** Potential risks and benefits of GMOs.

Dimension	Potential Benefits	Potential Risks	Key Question for Assessment
**Human health**	New medicines, disease models, xenotransplantation	Toxicity, allergenicity, zoonoses, immune reactions	Does the modification introduce a biologically relevant new hazard?
**Food security**	Increased productivity, disease resistance, environmental tolerance	Unintended changes in food composition	Is food from the modified animal as safe and nutritionally appropriate as the comparator?
**Animal health**	Disease resistance, heat tolerance	Unexpected physiological effects	Is the modified animal healthier or less healthy than its comparator?
**Animal welfare**	Elimination of painful procedures such as dehorning	Production traits could increase physiological stress	Does the modification improve or worsen the animal’s welfare?
**Environment**	Reduced chemical use, lower resource consumption, reduced disease transmission	Escape, gene flow, ecological disruption	What happens if the GM organism interacts with natural populations?
**Evolution**	Reduced pathogen burden	Selection for pathogen resistance or virulence	Could the modification alter pathogen evolution?
**Biodiversity**	Potential reduction in invasive or disease-vector species	Non-target effects and ecosystem changes	Could population-level intervention alter ecological networks?
**Medicine**	Replacement organs and therapeutic proteins	Infection, immune rejection and long-term uncertainty	Can risks be controlled throughout the lifetime of the recipient?
**Agriculture**	Faster genetic improvement than conventional breeding	Concentration of particular genotypes and reduced genetic diversity	Does technological improvement create new systemic vulnerabilities?

## Data Availability

No new data were created or analyzed in this study. Data sharing is not applicable to this article.
